# The Interactions of Airway Bacterial and Fungal Communities in Clinically Stable Asthma

**DOI:** 10.3389/fmicb.2020.01647

**Published:** 2020-07-21

**Authors:** Hai-yue Liu, Chun-xi Li, Zhen-yu Liang, Shi-yu Zhang, Wan-ying Yang, Yan-mei Ye, Yan-xia Lin, Rong-chang Chen, Hong-wei Zhou, Jin Su

**Affiliations:** ^1^Chronic Airways Diseases Laboratory, Department of Respiratory and Critical Care Medicine, Nanfang Hospital, Southern Medical University, Guangzhou, China; ^2^State Key Laboratory of Organ Failure Research, Microbiome Medicine Center, Division of Laboratory Medicine, Zhujiang Hospital, Southern Medical University, Guangzhou, China; ^3^State Key Laboratory of Respiratory Disease, National Clinical Research Center for Respiratory Disease, Guangzhou Institute of Respiratory Health, The First Affiliated Hospital of Guangzhou Medical University, Guangzhou, China

**Keywords:** 16S rRNA, airway microbiome, asthma, bacterial-fungal interactions, ITS

## Abstract

Dysbiotic airway microbiota play important roles in the inflammatory progression of asthma, and exploration of airway microbial interactions further elucidates asthma pathogenesis. However, little is known regarding the airway bacterial-fungal interactions in asthma patients. We conducted a cross-sectional survey of the sputum bacterial and fungal microbiota from 116 clinically stable asthma patients and 29 healthy controls using 16S rRNA gene and ITS1 sequencing. Compared with healthy individuals, asthma patients exhibited a significantly altered microbiota and increased bacterial and fungal alpha diversities in the airway. Microbial genera *Moraxella*, *Capnocytophaga*, and *Ralstonia* (bacteria) and *Schizophyllum*, *Candida*, and *Phialemoniopsis* (fungi) were more abundant in the asthma airways, while *Rothia*, *Veillonella* and *Leptotrichia* (bacteria) and *Meyerozyma* (fungus) were increased in healthy controls. The Moraxellaceae family and their genus *Moraxella* were significantly enriched in asthma patients compared with healthy controls (80.5-fold, *P* = 0.007 and 314.7-fold, *P* = 0.027, respectively). Moreover, Moraxellaceae, along with *Schizophyllum*, *Candida*, and *Aspergillus* (fungal genera), were positively associated with fungal alpha diversity. Correlation networks revealed 3 fungal genera (*Schizophyllum*, *Candida*, and *Aspergillus*) as important airway microbes in asthma that showed positive correlations with each other and multiple co-exclusions with other common microbiota. Moraxellaceae members were positively associated with asthma-enriched fungal taxa but negatively related to several healthy-enriched bacterial taxa. Collectively, our findings revealed an altered microbiota and complex microbial interactions in the airways of asthma patients. The Moraxellaceae family and their genus *Moraxella*, along with 3 important fungal taxa, showed significant interactions with the airway microbiota, providing potential insights into the novel pathogenic mechanisms of asthma.

## Introduction

Asthma is a hyperresponsive disease of the airway that affects more than 300 million people worldwide and has a continuously increasing prevalence (Lambrecht and Hammad, 2015). Two of the major pathogeneses of asthma are chronic airway inflammation and host immune responses (Lambrecht and Hammad, 2015; Weiss, 2017). Bacterial, fungal and viral infections induce airway inflammation and are associated with the host immune response in asthma (Sharpe et al., 2015; Lan et al., 2016). Recently, interest in the associations of the airway bacterial microbiota with host immune responses and chronic inflammation has been increasing (Ver Heul et al., 2018), and the airway fungal microbiota is becoming recognized as a factor correlated with host immune responses in asthma (Zhang et al., 2017).

Traditional culture-dependent studies have shown that colonization of the airway by pathogenic bacteria is associated with inflammation and with the severity and exacerbation of asthma (Wood et al., 2010; Zhang et al., 2012). Early colonization with *Moraxella catarrhalis*, *Haemophilus influenzae*, and/or *Streptococcus pneumoniae* has been associated with increased risks of subsequent wheezing and asthma (Bisgaard et al., 2007), and these bacteria contribute to acute wheeziness and exacerbation of asthma in young children (Bisgaard et al., 2010). Pathogenic bacteria, such as *Moraxella* spp., in the airway are related to increased asthma susceptibility and exacerbation of asthma by inducing inflammatory immune responses (Larsen et al., 2014), and *Moraxella* spp. are particularly closely related to asthma, as one of the dominant pathogenic species found in the airway bacterial community (Green et al., 2014).

With the advent of culture-independent techniques, human airways have been shown to harbor unique microbial communities (including bacteria, fungi and viruses) that are closely correlated with chronic respiratory diseases, including asthma (Jartti and Gern, 2017; Durack et al., 2018; Tipton et al., 2018). The airway bacterial community in asthma patients differs significantly from that in healthy individuals, exhibiting greater bacterial diversity, more Proteobacteria members (especially *Moraxella* spp.) and fewer Bacteroidetes members (Hilty et al., 2010; Zhang et al., 2016). The use of culture-independent techniques has also improved the understanding of the roles airway bacteria play in the risk, pathogenesis, and clinical presentation of asthma (Kozik and Huang, 2019). Several studies have shown that airway bacteria are important factors in the inception and development of asthma (Beigelman et al., 2014) and that they are correlated with disease-related features, severity and the therapeutic response in asthma (Green et al., 2014; Huang and Boushey, 2015; Li et al., 2017; Taylor et al., 2018; Sharma et al., 2019). Furthermore, the airway fungal community has been observed to be altered in asthma (van Woerden et al., 2013). Although most studies of the airway microbiome and asthma have focused on the bacterial microbiota, the airway fungal microbiota is also likely to have a significant impact on asthma (Nguyen et al., 2015). However, the airway fungal microbiota has not been well characterized using culture-independent techniques.

Within the context of complex poly-microbial communities, single-species microbial analyses may be insufficient because different microbial communities can interact with each other and affect pathogenesis (Peters et al., 2012). Airway bacterial-bacterial interactions are believed to be associated with immune responses and airway inflammation (Kyda et al., 2011), influencing the therapeutic response, disease progression and clinical outcome of lung diseases (Twomey et al., 2012; Short et al., 2014). Moreover, recent studies have provided insights into airway bacterial-fungal interactions, which may drive or exacerbate chronic airway inflammatory disease and contribute to decreased lung function (Nguyen et al., 2015; Zhang et al., 2017). The microbial interactions are not only influenced by the combination of microbiota, but also by the host and local environment, such as antibiotics and immune system (Krüger et al., 2019; Nogueira et al., 2019). For example, *Candida* spp. can overgrow under the condition of broad-spectrum antibiotics and immunosuppression (Chanda et al., 2017). However, microbial interactions, especially bacterial-fungal interactions, have not been extensively studied in asthma, and very little is known regarding the key/important microbial communities in the airway microbial interactions in asthma.

In this cross-sectional study, we aimed to explore the characteristics of microbiota and the complex interactions between microbial communities in the airway of clinically stable asthma patients using high-throughput sequencing methodologies. We tested the hypotheses that there may be key/important microbial communities that play an important role in the bacterial-fungal interactions associated with asthma.

## Materials and Methods

### Study Design and Subjects

This study was approved by the ethics committee of Southern Medical University (Permit No. 2012-072). All subjects provided written informed consent, in accordance with the Declaration of Helsinki. A total of 145 sputum samples were collected from 116 asthma patients and 29 healthy controls enrolled at Nanfang Hospital, Southern Medical University (Guangzhou, China), between June 2015 and December 2016. After sequencing, 7 samples with insufficient numbers of V4 sequences were excluded, samples from 138 of 145 participants were ultimately analyzed for bacterial community composition and total 145 samples were analyzed for fungal community composition. Clinical information collected from the subjects included age, sex, body mass index (BMI), forced expiratory volume in 1 s (FEV1), forced vital capacity (FVC), sputum eosinophils (%) and neutrophils (%), smoking history and use of inhaled corticosteroids (ICSs, Table 1). According to previous studies, the airway microbiome was related to inflammatory phenotypes of asthma (Sverrild et al., 2017; Pang et al., 2019; Sharma et al., 2019). Eosinophil and neutrophil subgroups were divided into: (1) EOS-low (eosinophils < 3%) and EOS-high (eosinophils ≥ 3%) groups, (2) NEU-low (neutrophils < 61%) and NEU-high (neutrophils ≥ 61%) groups, and (3) EOS-NEU-high (both eosinophils ≥ 3% and neutrophils ≥ 61%) and EOS/NEU-low (either eosinophils < 3% or neutrophils < 61%) groups according to the granulocyte count in induced sputum (Carr et al., 2018). Besides, we randomly selected 46 BMI-matched subjects (23 asthma patients and 23 healthy controls) for a sub-analysis to explore whether the airway microbial differences were related to BMI (Supplementary Table S1).

**TABLE 1:** Clinical characteristics of the subjects.

**Parameters**	**Healthy (*n* = 29)**	**Asthmatic (*n* = 116)**	*P*-value*
Age (years)	44.3 ± 23.1	42.7 ± 14.0	0.733
Sex, *n* (male/female)	21/8	62/54	0.065
BMI (kg/m^2^)	21.9 ± 4.0	23.7 ± 3.6	0.037
FEV1 pred (%)	–	87.9 ± 18.8	–
FVC pred (%)	–	101.9 ± 16.4	–
FEV1/FVC (%)	–	86.7 ± 14.6	–
Sputum eosinophil (%)	–	6.41 ± 13.93	–
Sputum neutrophil (%)	–	65.03 ± 23.04	–
Smoking, *n* (yes/no)	9/20	24/91	0.244
ICS, *n* (yes/no)	–	78/38	–

BMI: body mass index, FEV1: forced expiratory volume in 1 s, FVC: forced vital capacity, ICS: inhaled corticosteroids. *The data are presented as *n* (chi-square test) or as the mean ± standard deviation (independent *t*-test). *P* < 0.05 is considered significant between groups.

The inclusion criteria for asthma patients included age >15 years, initial diagnosis based on the Global Initiative for Asthma (GINA) guidelines (Accordini et al., 2011), and a positive bronchodilator reversibility test result (FEV1 increased by >12% and 200 mL after inhaling 400 mg of salbutamol) or a positive methacholine provocation test result. All subjects were free of clinical bacterial, fungal and viral infection at the time of the study. Exclusion criteria included respiratory tract infection diagnosed by chest X-ray (each patient underwent chest X-ray) within the past 4 weeks, the presence of any airway disease other than asthma, a peripheral white blood cell (WBC) count outside the normal range and antibiotic usage within 4 weeks of enrollment.

### Sample Collection, Processing and Sequencing of the Bacterial and Fungal Microbiota

For sputum induction and processing, the recommendations of the Task Force on Induced Sputum of the European Respiratory Society were followed (Guiot et al., 2017). All samples were immediately stored at −80°C for subsequent DNA extraction after collection. The sputum samples were thawed under ventilation for 15 min, and genomic DNA extraction was performed using the Total Genomic DNA Nucleic Acid Extraction Kit (Bioeasy Technology, Inc., China) according to the manufacturer’s instructions.

The V4 hypervariable region of the bacterial 16S rRNA gene and the internal transcribed spacer 1 (ITS1) region of the fungal 18S–28S rRNA genes were amplified using barcoded primers, and the amplicons were sequenced using the Ion Torrent platform (Ion PGM^TM^ Hi-Q^TM^ QT2 Kit). Detailed information on the 16S rRNA V4 regions and ITS1 genes amplification and purification steps was provided in our previous studies (Su et al., 2015). For bacteria, the V4 hypervariable region of the 16S rRNA gene was PCR amplified using the primers V4F (5′-GTGTGCCAGCMGCCGCGGTAA-3′) and V4R (5′-CCGGACTACHVGGTWTCTAAT-3′). For fungi, the ITS1 genes were amplified using the primers ITS1F (5′-CTTGGTCATTTAGAGGAAGTAA-3′) and ITS1R (5′-GCTGCGTTCTTCATCGATGC-3′). Each primer included Ion torrent sequencing adapters (forward primer, including the adapter: 5′-CCATCTCATCCCTGCGTGTCTCCGACTCAG-3′, reverse primer, including the adapter: 5′-CCTCTCTATGGGCAGTCGGTGAT-3′) and unique barcodes. For PCR amplification of the V4 hypervariable region of the bacterial 16S rRNA gene, the cycling conditions included an initial denaturation step at 94°C for 2 min, 30 cycles at 94°C for 30 s, 52°C for 30 s and 72°C for 30 s, and a final extension at 72°C for 5 min. The conditions for ITS1 gene PCR included an initial denaturation step at 94°C for 15 min, 5 cycles at 95°C for 30 s, 50°C for 30 s and 72°C for 1 min; 35 cycles at 95°C for 30 s, 65°C for 30 s and 72°C for 1 min; and a final extension step at 72°C for 15 min. PCR products were purified and the fragment with a length of approximately 300bp was retained using a DNA purification kit (Thermo Fisher Scientific, United States) before sequencing.

### Sequence Processing and Statistical Analysis

Sequence processing and analysis were performed using “Quantitative Insights into Microbial Ecology” (QIIME) 1.9.1 (Caporaso et al., 2010). First, the barcode primers were trimmed and filtered if they contained ambiguous reads or mismatches in the primer regions following the barcoded Illumina paired-end sequencing (BIPES) protocol (Zhou et al., 2011). Subsequently, we removed sequences that had more than one mismatch in 40-70-bp regions. Next, we screened and removed chimeras using UCHIME in *de novo* mode to obtain high-quality sequence reads of the 16S rRNA gene or ITS1 region (Edgar et al., 2011). In addition, the bioinformatics codes used for data processing are available from https://github.com/Haiyue123/Airway-microbiome. After quality filtering and chimera removal, 16S rRNA gene sequencing resulted in a median read depth of 13,262, and ITS1 DNA sequencing resulted in a median read depth of 5,936. Both the 16S rRNA V4 region and ITS1 DNA sequencing data of all subjects were normalized to a uniform depth of 2,000 reads based on rarefaction curve asymptotes and Good’s coverage values. A comparable rarefaction depth has been used in airway microbiome analyses (Gomez and Chanez, 2016; Taylor et al., 2018). Seven samples were excluded from the 16S V4 data analysis after normalization. The negative controls for DNA extraction and PCR steps were included in our sequencing run, and none of the bacterial or fungal OTUs presents in the reagent controls had >50 read counts.

The taxonomy of representative 16S rRNA gene sequences was determined using Python Nearest Alignment Space Termination (PyNAST) with the Greengenes 13_8 database as the reference, and multiple alignments of representative sequences were performed using PyNAST (Al-Hebshi et al., 2015). The taxonomy of representative ITS sequences was determined using the UNITE database (Nilsson et al., 2018). Representative 16S rRNA gene or ITS1 sequences were classified into specific taxa using the Ribosome Database Project (RDP) classifier (Whelan and Surette, 2017). The operational taxonomic units (OTUs) were assigned by clustering the reads with 97% sequence similarity using USEARCH (Westcott and Schloss, 2015). Briefly, the OTU representative sequences of the important OTUs (with relative abundances (RA) of >1% in at least one group) were BLASTn-searched (BLAST v2.5.0) against the non-redundant reference database (Supplementary Tables S2, S3). The sequences were deposited in the European Nucleotide Archive (ENA) under accession number PRJEB28853.

Alpha diversity (within-sample diversity) was evaluated using the following parameters: the Shannon index, which indicates the evenness and richness of the microbial community, and the observed OTUs index, which reflects the richness of species. Beta diversity (dissimilarity between samples) was calculated by principal coordinates analysis (PCoA) using Bray-Curtis distances, and statistical values were evaluated via the Adonis method. Differential features between groups were identified using linear discriminant analysis (LDA) effect size (LEfSe) with a threshold cut-off value of 2.0 for the logarithmic LDA score (Segata et al., 2011). We selected and presented the abundant taxa (phyla, families, genera and OTUs) with RA of >1% in at least one group for our subsequent analyses, which included Spearman rank correlation analysis and SparCC correlation analysis. Because of the relatively low reliability of the low abundant taxa and the insufficient analysis effect of few data, in order to view these microbial populations in numerous samples, it has been a standard protocol to show microbial communities with RA >1% (Schei et al., 2017; Stewart et al., 2017; Leung et al., 2018). Clinical characteristics were evaluated using IBM SPSS version 20.0, and figure s were generated using GraphPad Prism version 7.00 and R version 2.1.1. Network analysis using SparCC (*P* < 0.05) was performed in Cytoscape 3.7.2 (Friedman and Alm, 2012). For all statistical analyses, a *P*-value of <0.05 was considered statistically significant.

## Results

### Clinical Characteristics of the Subjects and Their Relationship With the Airway Microbiota

A total of 145 sputum samples were obtained from 116 asthma patients and 29 healthy controls. No significant differences were found based on age, sex or smoking history between the healthy and asthmatic groups, but asthma patients had a higher BMI than healthy controls (*P* = 0.037, Table 1). To identify the influence of BMI on airway microbiota, a subanalysis using 46 BMI-matched subjects (23 asthma patients and 23 healthy controls) was performed (Supplementary Table S1).

Then, we explored the relationship between the airway microbiota and the clinical features of asthma. For alpha diversity, we found that an increase in the eosinophil count was related to a decrease in fungal alpha diversity (Shannon index, *R* = −0.21, *P* = 0.06; observed OTUs index, *R* = −0.29, *P* < 0.05, Spearman rank test). Both the bacterial and fungal alpha diversity of the EOS-low group were higher than those of the EOS-high group (Bacteria: Shannon index, *P* = 0.067; observed OTUs index, *P* < 0.05. Fungi: Shannon index, *P* = 0.052, observed OTUs index, *P* < 0.01. Mann–Whitney *U*-test). In the EOS-NEU-high group, the observed OTUs index of fungal microbiota was lower than the EOS/NEU-low group (*P* = 0.026). For beta diversity, the FVC (%) was significantly associated with bacterial beta diversity (Bray-Curtis distance, Adonis, *R*^2^ = 0.015, *P* < 0.05). The EOS-low vs. EOS-high groups, the ICS vs. non-ICS groups and the EOS-NEU-high vs. EOS/NEU-low group exhibited distinct fungal beta diversities (Bray-Curtis distance, Adonis, *R*^2^ = 0.027, *P* < 0.01, *R*^2^ = 0.022, *P* < 0.05, and *R*^2^ = 0.028, *P* < 0.05, respectively).

For subsequent analyses, we included 15 dominant bacterial families and 4 dominant fungal families, with 14 dominant bacterial genera and 4 dominant fungal genera (with average RA of >1% in any group, Supplementary Figures S1B, S2B). Spearman analysis showed no association between most of the bacterial or fungal microbiota and clinical features of asthma, except for a few bacterial taxa associated with some clinical parameters. For example, the Prevotellaceae family and their genus *Prevotella* were negatively related to FEV1 (%) (*R*^2^ = −0.22, *P* < 0.05), while the Peptostreptococcaceae family and their genus *Peptostreptococcus* showed positive associations with both FEV1 (%) (*R*^2^ = 0.23, *P* < 0.05 and *R*^2^ = 0.19, *P* < 0.05, respectively) and FVC (%) (*R*^2^ = 0.35, *P* < 0.001 and *R*^2^ = 0.29, *P* < 0.01, respectively). Moreover, the Porphyromonadaceae family and their genus *Porphyromonas* were positively associated with neutrophils (*R*^2^ = 0.22, *P* < 0.05), whereas the genus *Haemophilus* was negatively associated neutrophils (*R*^2^ = −0.21, *P* < 0.05) and the genus *Meyerozyma* showed positive correlations with eosinophils (*R*^2^ = 0.20, *P* < 0.05). Streptococcaceae family and their genus *Streptococcus* were reduced (both *P* < 0.05), while Debaryomycetaceae family and their genus *Meyerozyma* were enriched (both *P* < 0.05) in EOS-NEU-high group compared with EOS/NEU-low group.

### Airway Bacterial and Fungal Communities in Asthma Patients Differ From Those in Healthy Individuals

**FIGURE 1:**
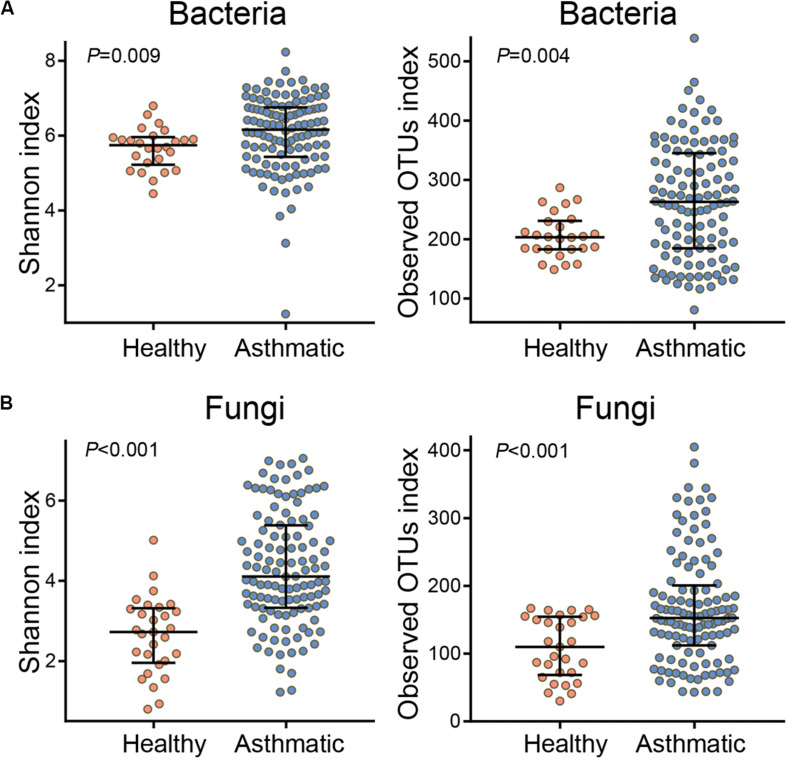
Alpha diversity of the bacterial **(A)** and fungal **(B)** communities in healthy individuals and asthma patients. The Shannon index indicates the evenness and richness of diversity of the airway microbial community, and the observed OTUs index reflects the richness of the airway microbial community. *P*-values were determined using the Wilcoxon rank-sum test, and *P* < 0.05 indicates statistical significance between groups.

Compared with healthy individuals, asthma patients showed a significantly high airway alpha diversity (calculated using the Shannon index and observed OTUs index, Figure 1) and distinct differences in the beta diversity (Supplementary Figures S1A, S2A) of both the bacterial and fungal communities. In order to explore the certain relationship between BMI and airway microbiota, we attempted to find the community differences of airway microbiota based on BMI status in asthma patients. The alpha and beta diversity of both bacterial and fungal communities showed that airway microbial composition was not markedly associated with BMI status in asthma patients (Supplementary Figure S3). Moreover, the sub-analysis performed on 46 BMI-matched subjects also indicated that the observed microbial differences were not related to BMI (Supplementary Figures S4, S5).

In addition, we compared the airway bacterial and fungal community compositions between healthy individuals and asthma patients (Supplementary Figures S1, S2). Of the 413 bacterial genera identified in asthma patients, the most abundant genera were *Prevotella* (16.1%), *Streptococcus* (14.6%), *Neisseria* (12.7%), *Porphyromonas* (8.9%), and *Haemophilus* (5.8%). Of the 384 fungal genera identified in asthma, the most abundant genera were *Meyerozyma* (25.4%), *Schizophyllum* (2.3%), *Aspergillus* (2.0%) and *Candida* (1.5%). According to the UNITE database, an average of 14.56% fungi were annotated as “unidentified fungus” in our data. LEfSe analysis showed microbial differences in the family level and its genus level between the two groups; specifically, Micrococcaceae/*Rothia*, Lachnospiraceae, Veillonellaceae/*Veillonella*, Leptotrichiaceae/ *Leptotrichia*, Burkholderiaceae (bacteria) and Debaryomyce- taceae/*Meyerozyma* (fungi) were enriched in healthy subjects, while the abundances of Flavobacteriaceae/*Capnocytophaga*, *Ralstonia*, Moraxellaceae/*Moraxella* (bacteria), Schizophyllaceae/ *Schizophyllum*, Saccharomycetaceae/*Candida* and Sordariaceae/ *Phialemoniopsis* (fungi) were higher in asthma patients.

Further comparison of the OTUs revealed that several bacterial taxa were highly abundant in healthy individuals: otu4 (*Gemellaceae* sp.), otu12 (*Porphyromonas* sp.), otu53 (*Rothia mucilaginosa*), otu13 (*Streptococcus* sp.) and otu22 (*Veillonella parvula*). However, significantly increased abundances of otu19 (*Moraxella* sp.), otu3 (*Porphyromonas* sp.) and otu21 (*Prevotella* sp.) were observed in asthma patients. In addition, 7 fungal OTUs were increased in asthma patients, including otu2 (*Candida* sp.), otu3 (*Sordariomycetes* sp.), otu4 (*Candida albicans*), otu6 (*Schizophyllum commune*), otu9 (*Aspergillus niger*), otu12 (*Malassezia restricta*) and otu157 (*Agaricomycetes* sp.) (Supplementary Tables S4, S5).

**FIGURE 2:**
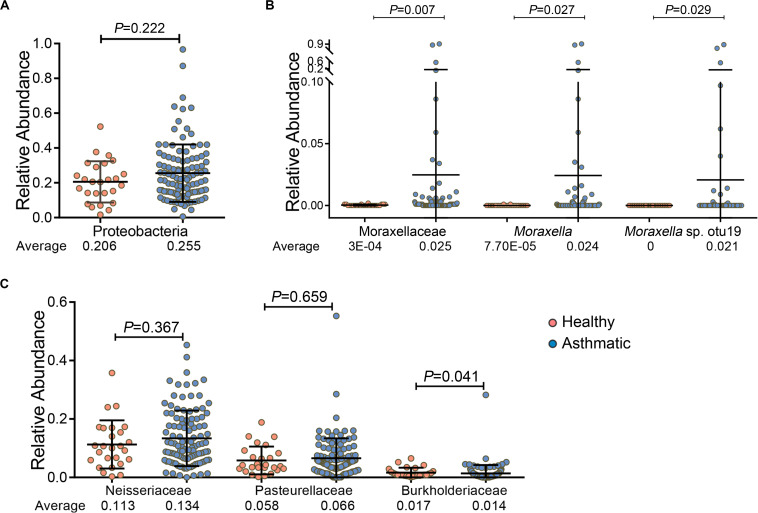
Comparison of the RA of phylum Proteobacteria **(A)**, the Moraxellaceae family and their genus *Moraxella* and *Moraxella* sp. otu19 **(B)**, and other families belonging to Proteobacteria (except Moraxellaceae) **(C)** between healthy individuals and asthma patients. *P*-values (Wilcoxon rank-sum test) and average RA are presented. *P* < 0.05 indicates statistical significance between groups.

In particular, we found that among the 5 major bacterial phyla, Proteobacteria was relatively more abundant in asthma patients than in healthy controls, although the increase was not statistically significant (*P* = 0.222, Figure 2A). However, the Moraxellaceae family and their genus *Moraxella* and *Moraxella* sp. otu19, which belong to Proteobacteria, were significantly more abundant in the airways of asthma patients than in those of healthy individuals. Specifically, the RA of the Moraxellaceae family and their genus *Moraxella* in asthma patients were 80.5- and 314.7-fold higher, respectively, than those in healthy individuals, and *Moraxella* sp. otu19 was detected only in asthma patients (Figure 2B). However, the abundances of other families belonging to Proteobacteria, such as Neisseriaceae and Pasteurellaceae, were not significantly increased; and some were even decreased, for example, the abundance of Burkholderiaceae was relatively low in asthma patients (*P* = 0.041, Figure 2C).

### Relationships Among Airway Bacterial/Fungal Communities and Airway Fungal Diversity

As shown above, the airway microbial diversity in asthma patients differed from that in healthy individuals. Thus, we explored whether there was a relationship among the discriminated taxa and airway bacterial/fungal alpha diversity in asthma patients.

**FIGURE 3:**
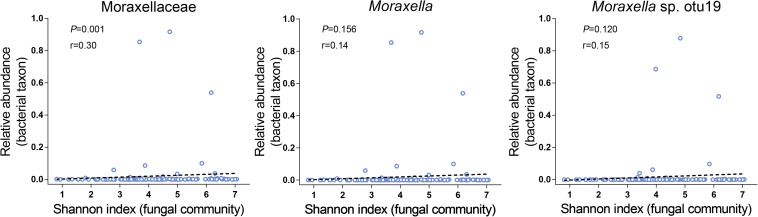
Relationships between the Moraxellaceae family and their genus *Moraxella* and *Moraxella* sp. otu19 and fungal alpha diversity. The *X*-axis of each panel represents the Shannon index values of the fungal community, and the *Y*-axis represents the RA of each bacterial taxon. R (cut-off of greater than 0.3) and *P*-values (*P* < 0.05 indicates statistical significance) are presented using Spearman rank correlation analysis.

We first focused on the bacterial diversity and found no significant relationships between Proteobacteria, the Moraxellaceae family and their genus *Moraxella* and *Moraxella* sp. otu19 and bacterial diversity. However, a significantly positive correlation was found between Moraxellaceae and fungal diversity (*P* = 0.001, Figure 3). Next, we investigated whether the airway fungal community was related to fungal diversity and found that Schizophyllaceae, Saccharomycetaceae and Trichocomaceae (family level), *Schizophyllum*, *Candida*, *Aspergillus* (genus level), and *Candida* sp. otu2, *Aspergillus* sp. otu5, *Schizophyllum commune* otu6, *Aspergillus niger* otu9 and *Malassezia restricta* otu12 (OTU level) were positively correlated with fungal diversity, whereas *Meyerozyma guilliermondii* otu0 was negatively correlated (all *P* < 0.001). These findings indicate that Moraxellaceae and the abovementioned fungal taxa may play an important role in the disturbance of microbial community diversity.

### Airway Bacterial-Fungal Interactions of Asthma

**FIGURE 4:**
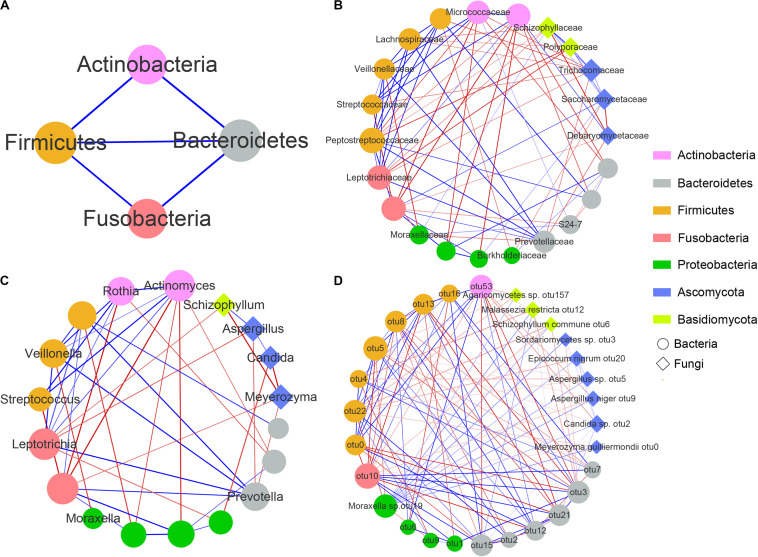
Correlation networks between airway microbial communities at the phylum **(A)**, family **(B)**, genus **(C)**, and OTU **(D)** levels. Each node represents a microbial taxon. The node shape denotes the microbe type (round, bacteria; diamond, fungus), and the node size represents the degree of connectivity. The microbial communities are colored by the phylum level. Red and blue edges represent negative and positive correlations, respectively (determined using SparCC). The thickness of the edges represents the *P*-values, with lower *P*-values represented by thicker lines.

To explore interactions among members of the airway bacterial and fungal microbiota in asthma patients, we used SparCC to build correlation networks of abundant taxa at the phylum, family, genus and OTU levels among 112 asthma patients who were analyzed for both bacterial and fungal communities (Figure 4).

The complex network revealed a few important microbial taxa that were closely connected with other portions of the airway microbial community. First, at the phylum level, Firmicutes, Bacteroidetes, Actinobacteria and Fusobacteria shared positive associations with each other. Second, at the family level, Moraxellaceae showed 5 negative correlations with bacterial families, including Leptotrichiaceae, Veillonellaceae and Prevotellaceae, but 2 positive correlations with Schizophyllaceae and S24-7. Moreover, we observed that 4 fungal families, namely, Schizophyllaceae, Saccharomycetaceae, Trichocomaceae and Polyporaceae exhibited strong positive correlations with each other, whereas they showed several negative correlations with families enriched in the airways of healthy controls, such as Lachnospiraceae, Leptotrichiaceae, Micrococcaceae, Burkholderiaceae and Debaryomycetaceae (Supplementary Figures S1, S2). At the genus level, only one edge was connected with *Moraxella*, which was negatively related to *Veillonella* (increased in healthy controls). In addition, both *Schizophyllum* and *Candida* showed a negative association with *Meyerozyma* but a positive association with *Aspergillus*. Finally, at the OTU level, *Moraxella* sp. otu19 was significantly positively associated with 10 fungal OTUs, including *Meyerozyma guilliermondii* otu0, *Malassezia restricta* otu12, *Agaricomycetes* otu157, *Candida* sp. otu2, *Epicoccum nigrum* otu20, *Sordariomycetes* sp. otu3, *Aspergillus* sp. otu5, *Schizophyllum commune* otu6 and *Aspergillus niger* otu9, most of which were increased in asthma patients (Supplementary Table S4). Moreover, both *Candida* sp. otu2 and *Aspergillus niger* otu9 were negatively associated with *Rothia mucilaginosa* otu53, and both *Aspergillus* sp. otu5 and *Schizophyllum commune* otu6 were negatively associated with *Veillonella parvula* otu22. However, no connections were found among *Candida* sp. otu2, *Sordariomycetes* sp. otu3, *Aspergillus* sp. otu5, *Schizophyllum commune* otu6 and *Aspergillus niger* otu9. Supplementary Table S6 showed the interaction networks between airway bacterial/fungal microbiota and the genus *Moraxella* and *Moraxella* sp. otu19 for asthma and healthy subjects.

In summary, these results suggested complex and close bacterial-fungal interactions in the airways of asthma patients. We found that the Moraxellaceae family and their genus *Moraxella* and *Moraxella* sp. otu19 exhibited multiple associations with other airway microbiota, and fungi from 3 genera *Schizophyllum*, *Candida* and *Aspergillus* showed close interactions with each other and the other airway microbiota. *Moraxella* spp. were positively associated with these asthma-enriched fungal taxa and negatively related to several healthy-enriched bacterial taxa. In addition, there were significantly positive correlations among the fungal families Schizophyllaceae, Saccharomycetaceae and Trichocomaceae, as well as among the fungal genera *Schizophyllum*, *Candida* and *Aspergillus*. These fungal taxa were negatively associated with multiple commensal airway microbiota, such as the family Burkholderiaceae and genus *Meyerozyma*.

## Discussion

Asthma is a chronic airway inflammatory disease associated with altered microbial communities in the airway, and these communities are closely related to airway inflammation. Consistent with previous studies, we observed higher bacterial alpha diversity in asthma patients than in healthy controls (Huang et al., 2011; Marri et al., 2013). In addition, the beta diversities (community composition) of both bacteria and fungi were different between asthmatic and healthy groups.

Recent studies showed that the airway microbiota is associated with disease-related features of asthma, such as BMI, FEV1 (%), and Asthma Control Questionnaire (ACQ) scores (Green et al., 2014; Huang et al., 2015). Our data showed correlation relationships between both FEV1 (%) and FVC (%) and a few bacterial taxa, including *Prevotella* spp. (negatively correlated) and *Peptostreptococcus* spp. (positively correlated), indicating that these taxa may be related to the disease severity of asthma. In accordance with previous studies, we found differences in the airway microbiota between different asthma phenotypes. Patients with EOS-low asthma had higher bacterial and fungal diversities and exhibited different Bray-Curtis distances than those with EOS-high asthma (Sverrild et al., 2017; Sharma et al., 2019). This pattern may suggest that the increased eosinophilic inflammation may interact with the increased airway microbial alpha diversity.

The airway microbiome is believed to shape airway inflammatory responses and impact disease outcomes (Kozik and Huang, 2019). In this study, we demonstrated positive correlations between both the Moraxellaceae family and their genus *Moraxella* and airway fungal alpha diversity. These findings suggest that the Moraxellaceae family and their genus *Moraxella* are associated with airway fungal communities in asthma patients. *Moraxella* spp. is widely recognized as a respiratory tract pathogen and is associated with several respiratory diseases, such as asthma, altering the disease susceptibility and severity (Bisgaard et al., 2010; Di Cicco et al., 2018; McCauley et al., 2019). Multiple studies have reported an altered airway microbial dysbiosis, with an increased abundance of Proteobacteria in asthma patients (Hilty et al., 2010; Huang et al., 2011, 2015; Goleva et al., 2013; Marri et al., 2013). Proteobacteria are a group of Gram-negative bacteria, with some genera notably known as pathogens. Previous study showed an overrepresentation of *Escherichia coli* in asthma patients compared with controls (Castro-Nallar et al., 2015), and a relationship between *Escherichia coli* bloodstream infection and asthma (Bang et al., 2013). Hilty et al. found that members of the phylum Proteobacteria, particularly *Haemophilus* and *Moraxella* spp., were significantly increased in asthma and COPD patients compared with controls (Hilty et al., 2010). Marri et al. observed an increased abundance of Gammaproteobacteria in asthma patients (Marri et al., 2013). The greater number of members from Proteobacteria supports the role of these bacteria in the development of asthma (Bisgaard et al., 2007). In this study, we found that the increase in Proteobacteria abundance could be partially attributed to significant increases in abundances of the Moraxellaceae family and their genus *Moraxella*, which in asthma patients were 80.5- and 314.7-fold higher, respectively, than those observed in healthy individuals. However, other families of Proteobacteria, such as Neisseriaceae and Pasteurellaceae, were not significantly increased; even Burkholderiaceae was decreased in asthma patients compared with healthy controls. It is generally known that there are complex interactions between the human immune system and microbiome, and the two are affected by each other (Belkaid and Harrison, 2017). For example, high levels of immunoglobulin A (IgA) can induce low-grade immune responses to allow the colonization of commensal bacteria. In contrast, IgA can also neutralize the toxins produced by microbiota and prevent the microbiota from adhering to intestinal mucosa (Vemuri et al., 2018). A previous study showed that there was a lack of Pasteurellaceae in wheezing infants compared with healthy controls (Cardenas et al., 2012). Moreover, Burkholderiaceae was found to be significantly reduced in the lungs of patients with rheumatoid arthritis, a common autoimmune disease. Therefore, we suspect that the decrease in these bacteria may be related to the immune system in asthma patients. However, more studies are needed to confirm these results.

In the present study, 3 fungal genera (*Schizophyllum*, *Candida* and *Aspergillus*) showed the closest association with asthma based on their series of correlation relationships in the networks. Among these genera, *Schizophyllum* and *Candida* were identified as being increased in asthma. *Schizophyllum* can cause a range of respiratory diseases in humans. According to a previous study, *Schizophyllum* spp. appears to enhance both the severity and exacerbation frequency of asthma (Ogawa et al., 2011); sensitization to *Schizophyllum* spp. is an important risk factor affecting exacerbation frequency and causing a rapid decline in lung function in asthma (Ogawa et al., 2013). Additionally, *Candida* spp. are considered to be pathogenically important in patients with asthma and has the highest detection rate in asthma children with fungal infection (Khosravi et al., 2009; Liu et al., 2019). Sensitization to *Candida* spp. is reported to be associated with severe asthma (Masaki et al., 2017). Another species of *Aspergillus* spp., well-known fungal pathogens of the respiratory tract, also plays an important role in asthma. *Aspergillus* spp. are associated with an increased risk for and exacerbation of asthma (Sharpe et al., 2015) and may contribute to severe asthma (Takazono and Sheppard, 2017). In addition, allergic bronchopulmonary aspergillosis (ABPA), which is the most severe allergic pulmonary disease and often occurs in patients with asthma, is caused by *Aspergillus* spp. Recently, culture-independent technologies have also revealed that *Aspergillus* spp., *Candida* spp., *Malassezia* spp. and *Schizophyllum* spp. were the dominant fungi in the airways of patients with chronic respiratory diseases, including chronic obstructive pulmonary disease (COPD), asthma, cystic fibrosis (CF) and bronchiectasis (Mac Aogain et al., 2018; Weaver et al., 2019).

Previous studies have highlighted the importance of complex microbial interactions in asthma (Kyda et al., 2011; Short et al., 2014), which may have dramatic effects on airway inflammation and disease outcome (Nguyen et al., 2015; Guiot et al., 2017; Chen et al., 2018). Our results suggested the presence of complex microbial networks in the airway of asthma patients. We found that *Moraxella* spp. and the fungal families Schizophyllaceae, Saccharomycetaceae and Trichocomaceae, as well as the fungal genera *Schizophyllum*, *Candida* and *Aspergillus*, were negatively associated with multiple healthy-enriched airway microbiota. Moreover, *Malassezia* species, common commensals of human skin, are associated with atopic conditions, such as atopic dermatitis, via the production of complex allergens (Gaitanis et al., 2012). van Woerden et al. found that *Malassezia* spp. were increased in the sputum of asthma patients, but they did not confirm the potential significance of this fungi (van Woerden et al., 2013). In this study, *Malassezia restricta* otu12 was more abundant in asthma patients, showing a positive association with fungal alpha diversity and *Moraxella* sp. otu19. Overall, our findings suggested that microbiota from the genera *Moraxella*, *Schizophyllum*, *Candida* and *Aspergillus* are associated with dysbiosis of airway fungal communities, and may play important roles in the airway microbiome via interactions with the airway mycobiome.

Our study is strengthened by the relatively adequate sample size of Chinese subjects (Zhang et al., 2016; Li et al., 2017) and by the combined exploration of airway bacterial and fungal communities. A major limiting factor is the observational study that provides only the possible relationships between *Moraxella*, *Schizophyllum*, *Candida* and *Aspergillus* (genera) and the airway microbiota. Another limiting factor was the relatively small size of healthy controls and the imbalanced gender ratio between healthy controls and asthmatic patients. In addition, the limited sample availability in our study prevented us from performing additional qPCR analysis. However, there may be differences in the absolute amount if we performed quantitative analysis. Therefore, future *in vivo* and vitro experiments are required to confirm the potential mechanism linking these microorganisms of interest with the host. A further limitation is the average of 14.56% unidentified reads of the ITS fungal sequences in our study, indicating the limited sequence availability in fungal databases according to previous studies (Mac Aogain et al., 2018; Weaver et al., 2019). However, most of the fungi that we considered have been effectively classified. In this study, 20% of the common fungal OTUs were unidentified at the genus level, including *Sordariomycetes* sp. otu3 and *Agaricomycetes* sp. otu157, which showed negative correlations with *Moraxella* sp. otu19 and several connections with other OTUs in the asthma airways. Although the unidentified fungal OTUs limited the comprehensive and rigorous interpretation of our results, there were still 80% of the common OTUs identified, providing relatively effective discovery for us. Therefore, a microbial database with more effective classification may help us to find more interesting and meaningful results.

In summary, we found an altered microbiota and characterized the complex bacterial-fungal interactions in the airways of asthma patients. The Moraxellaceae family and their genus *Moraxella*, along with 3 important fungal taxa, showed significant interactions with the airway microbiota, providing potential insights into the novel pathogenic mechanisms of asthma.

## Data Availability Statement

The datasets generated for this study can be found in the NCBI under accession number PRJEB28853.

## Ethics Statement

The studies involving human participants were reviewed and approved by 2012-072. Written informed consent to participate in this study was provided by the participants’ legal guardian/next of kin. Written informed consent was obtained from the individual(s), and minor(s)’ legal guardian/next of kin, for the publication of any potentially identifiable images or data included in this article.

## Author Contributions

HL, CL, HZ, and JS designed the experiments. HL, CL, ZL, SZ, YY, WY, RC, and YL collected the samples and performed the experiments. HL, CL, and ZL analyzed the data. HL, CL, HZ, and JS prepared the manuscript and had primary responsibility for its final content. All authors contributed to the article and approved the submitted version.

## Conflict of Interest

The authors declare that the research was conducted in the absence of any commercial or financial relationships that could be construed as a potential conflict of interest.

## Supplementary Material

FIGURE S1 The airway bacterial community composition between all asthmatic and healthy individuals. **(A)** Principal coordinates analysis (beta diversity) based on the Bray-Curtis distance for each sample of the bacterial community between healthy controls and asthma patients. **(B)** Bar plot of the most abundant bacterial microbiota at the genus level in healthy subjects and asthma patients (genera with average RA >1% in any group are shown). **(C)** LEfSe analysis results showing the differentially abundant bacterial taxa between the airway microbiota of healthy subjects and asthma patients.

FIGURE S2 The airway fungal community composition between all asthmatic and healthy individuals. **(A)** Principal coordinates analysis (beta diversity) based on the Bray-Curtis distance for each sample of the fungal community between healthy controls and asthma patients. **(B)** Bar plot of the most abundant fungal microbiota at the genus level in healthy subjects and asthma patients (genera with average RA >1% in any group are shown). **(C)** LEfSe analysis results showing the differentially abundant fungal taxa between the airway microbiota of healthy subjects and asthma patients.

FIGURE S3 Airway microbial community composition based on BMI status. **(A)** Alpha diversity (Shannon index and observed OTUs index) and **(B)** beta diversity (Bray-Curtis distance) of the bacterial and fungal microbial communities.

FIGURE S4 The airway bacterial community composition between BMI-matched asthma patients (*n* = 23) and healthy controls (*n* = 23). Alpha diversity (Shannon index and observed OTUs index, **A**), beta diversity (Bray-Curtis distance, **C**) and LEfSe results **(B)** of the bacterial community in healthy controls and asthma patients are shown.

FIGURE S5 The airway fungal community composition between BMI-matched asthma patients (*n* = 23) and healthy controls (*n* = 23). Alpha diversity (Shannon index and observed OTUs index, **A**), beta diversity (Bray-Curtis distance, **C**) and LEfSe results **(B)** of the fungal community in healthy controls and asthma patients are shown.

TABLE S1 Clinical characteristics of the 46 BMI-matched subgroups (23 healthy controls and 23 asthma patients).

TABLE S2 The BLAST results of the most abundant OTUs (average RA of >1% in any group) in the airway bacterial microbiota of healthy controls and asthma patients.

TABLE S3 The BLAST results of the most abundant OTUs (average RA of >1% in any group) in the airway fungal microbiota of healthy controls and asthma patients.

TABLE S4 Prevalence (P%) and RA (%) of the most abundant OTUs (RA of >1% in any group) in the airway bacterial microbiota of healthy controls and asthma patients.

TABLE S5 Prevalence (P%) and RA (%) of the most abundant OTUs (RA of >1% in any group) in the airway fungal microbiota of healthy controls and asthma patients.

TABLE S6 The interactions between airway bacterial/fungal microbiota and the genus *Moraxella* and *Moraxella* sp. otu19 in the networks for asthma and healthy subjects.
