# Supplementary figures and images for: The Interactions of Airway Bacterial and Fungal Communities in Clinically Stable Asthma
Source: Front Microbiol. 2020 Jul 21;11:1647. doi: 10.3389/fmicb.2020.01647 (PMC7396634; doi:10.3389/fmicb.2020.01647)

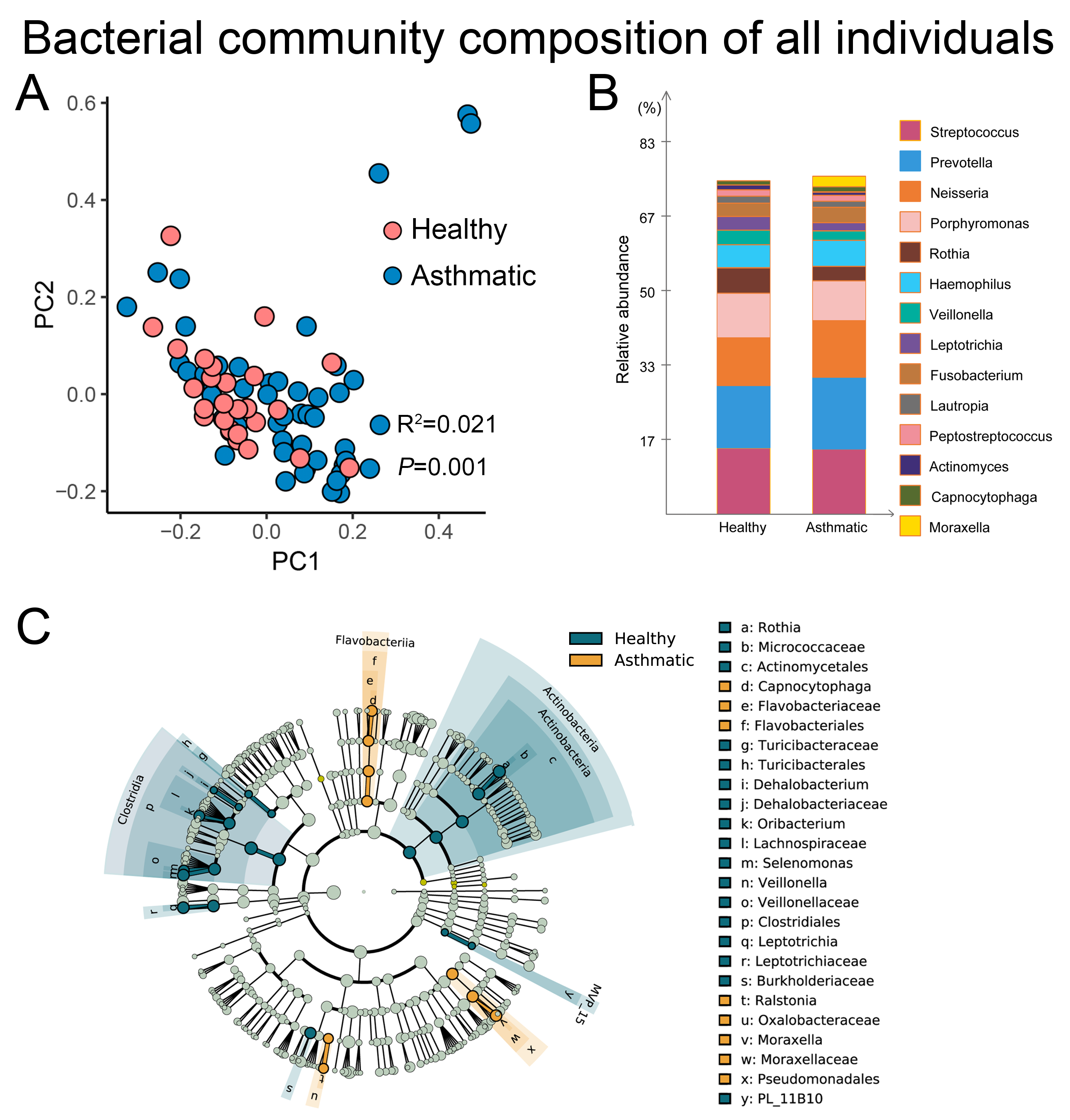

Supplement: FIGURE S1 — The airway bacterial community composition between all asthmatic and healthy individuals. (A) Principal coordinates analysis (beta diversity) based on the Bray-Curtis distance for each sample of the bacterial community between healthy controls and asthma patients. (B) Bar plot of the most abundant bacterial microbiota at the genus level in healthy subjects and asthma patients (genera with average RA >1% in any group are shown). (C) LEfSe analysis results showing the differentially abundant bacterial taxa between the airway microbiota of healthy subjects and asthma patients. [file Image_1.TIF]

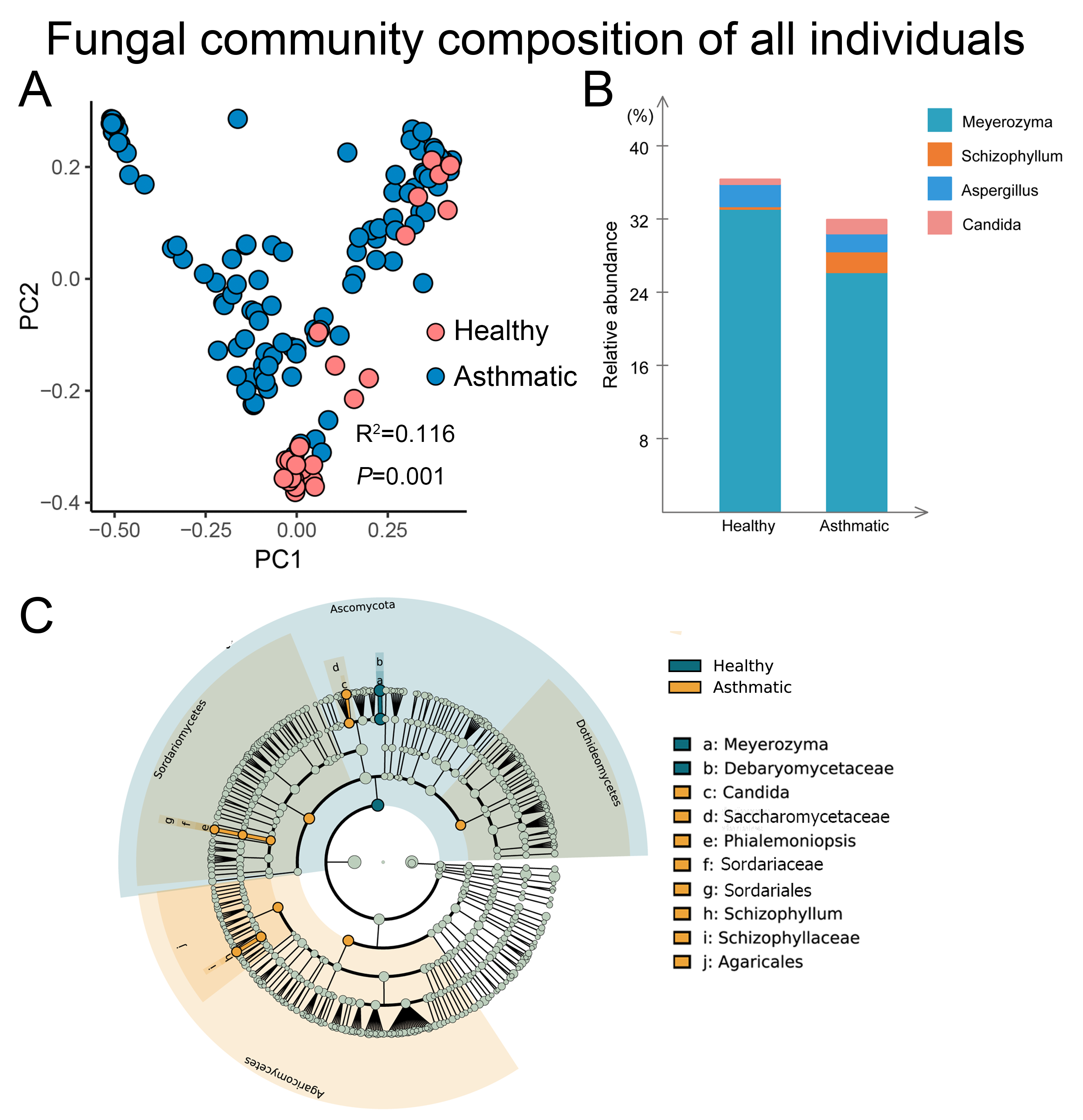

Supplement: FIGURE S2 — The airway fungal community composition between all asthmatic and healthy individuals. (A) Principal coordinates analysis (beta diversity) based on the Bray-Curtis distance for each sample of the fungal community between healthy controls and asthma patients. (B) Bar plot of the most abundant fungal microbiota at the genus level in healthy subjects and asthma patients (genera with average RA >1% in any group are shown). (C) LEfSe analysis results showing the differentially abundant fungal taxa between the airway microbiota of healthy subjects and asthma patients. [file Image_2.TIF]

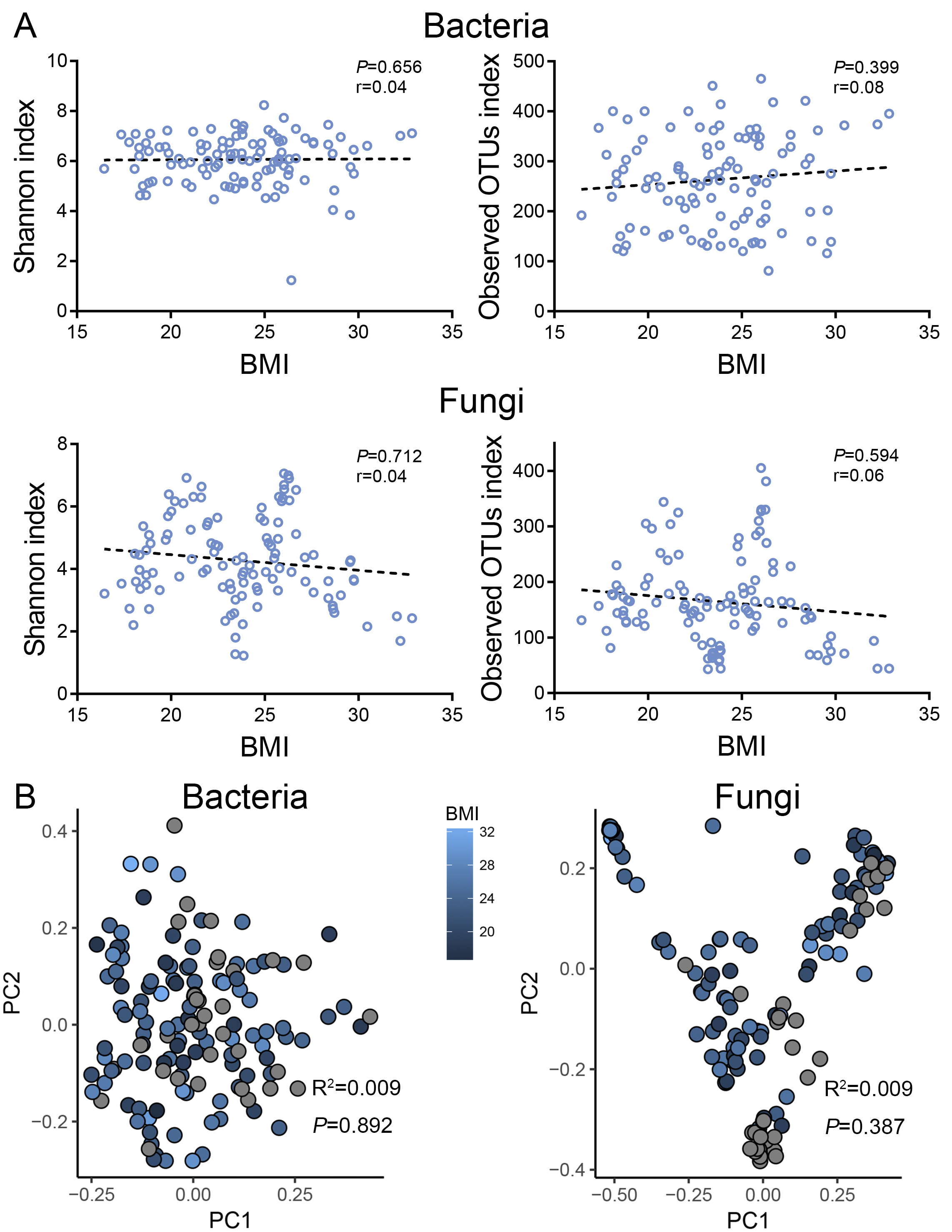

Supplement: FIGURE S3 — Airway microbial community composition based on BMI status. (A) Alpha diversity (Shannon index and observed OTUs index) and (B) beta diversity (Bray-Curtis distance) of the bacterial and fungal microbial communities. [file Image_3.TIF]

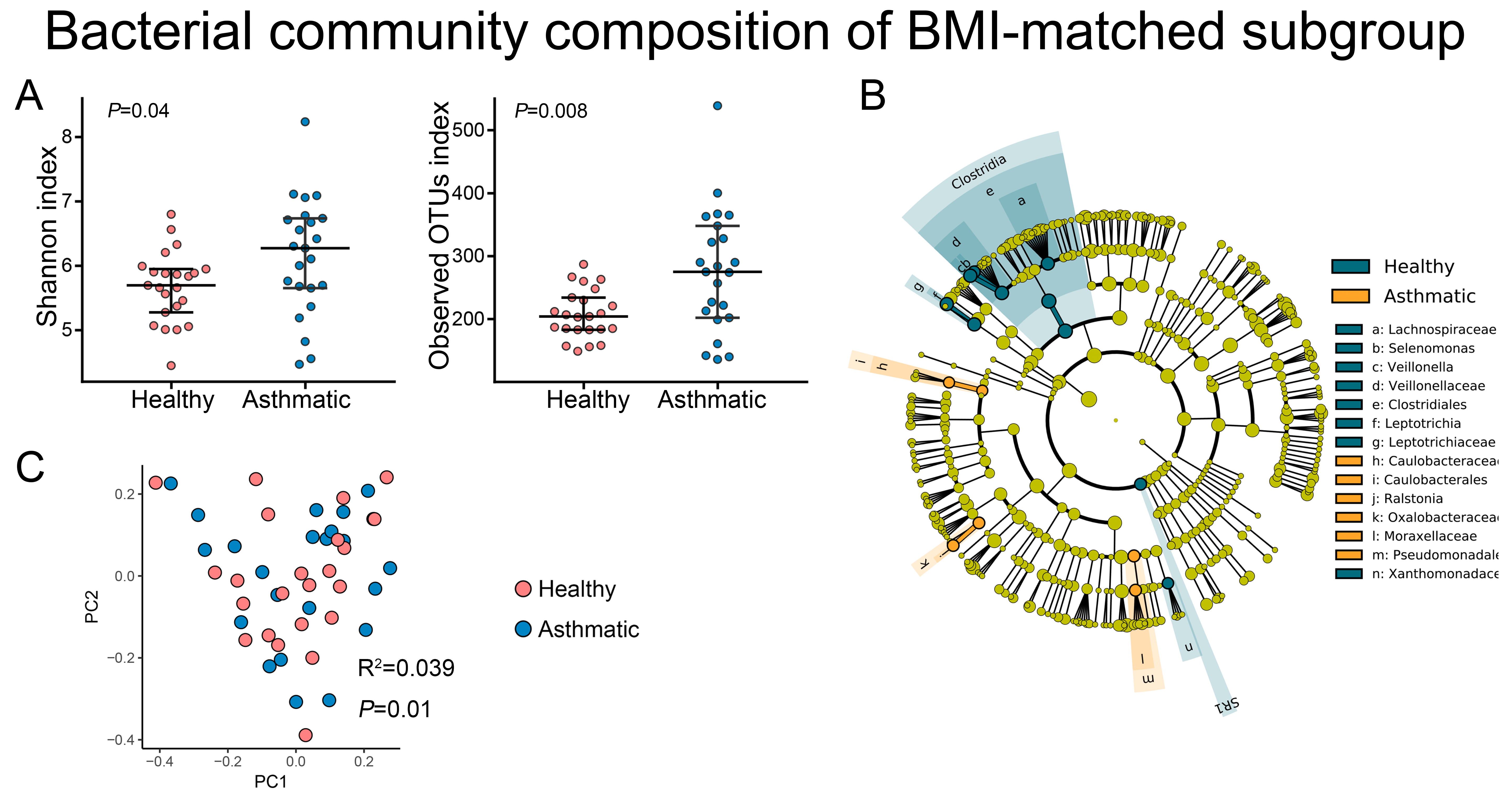

Supplement: FIGURE S4 — The airway bacterial community composition between BMI-matched asthma patients (n = 23) and healthy controls (n = 23). Alpha diversity (Shannon index and observed OTUs index, A), beta diversity (Bray-Curtis distance, C) and LEfSe results (B) of the bacterial community in healthy controls and asthma patients are shown. [file Image_4.TIF]

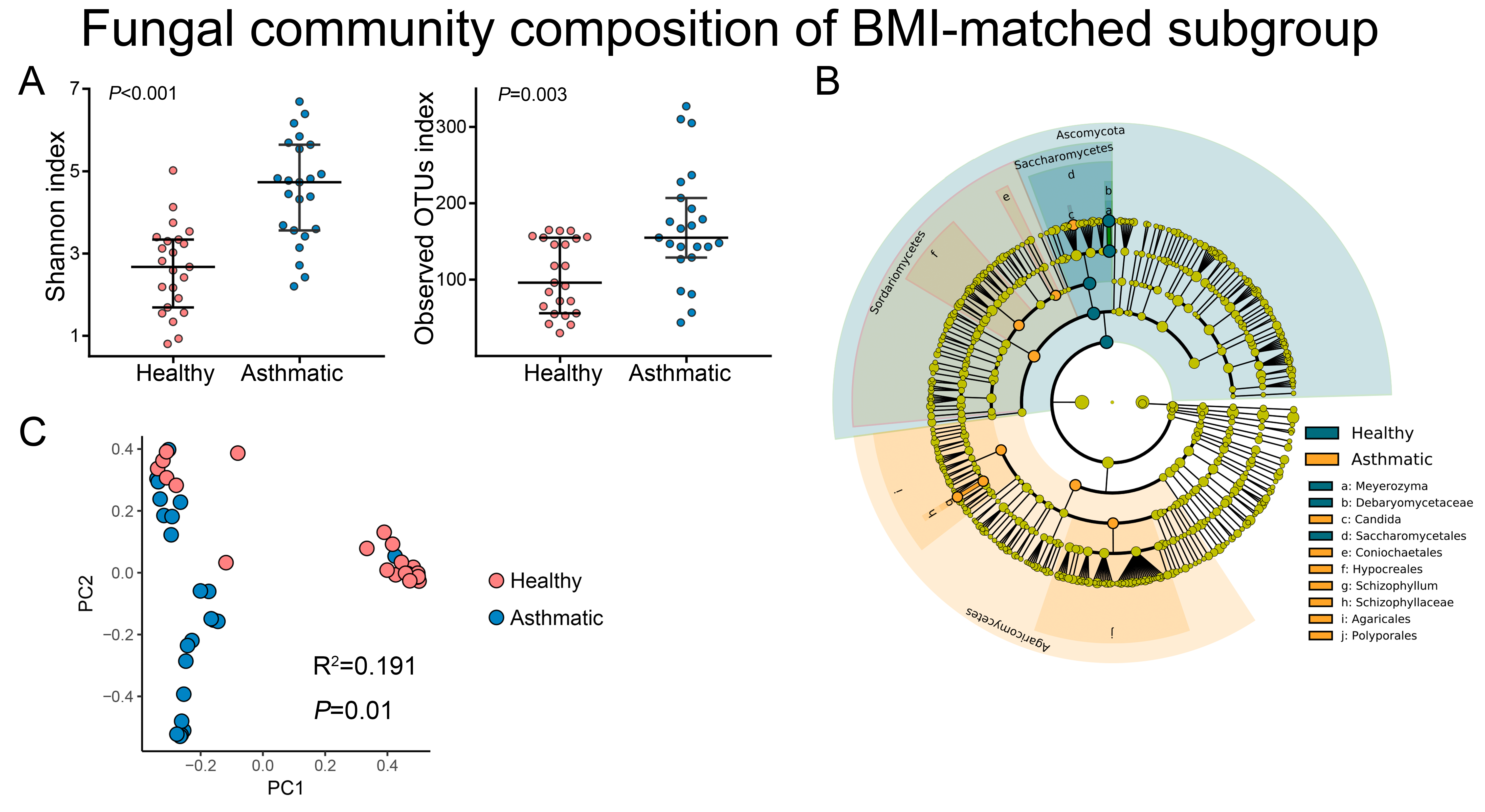

Supplement: FIGURE S5 — The airway fungal community composition between BMI-matched asthma patients (n = 23) and healthy controls (n = 23). Alpha diversity (Shannon index and observed OTUs index, A), beta diversity (Bray-Curtis distance, C) and LEfSe results (B) of the fungal community in healthy controls and asthma patients are shown. [file Image_5.TIF]
