# Supplementary material for: The Interactions of Airway Bacterial and Fungal Communities in Clinically Stable Asthma
Source: Front Microbiol. 2020 Jul 21;11:1647. doi: 10.3389/fmicb.2020.01647 (PMC7396634; doi:10.3389/fmicb.2020.01647)
Supplement: TABLE S1 — Clinical characteristics of the 46 BMI-matched subgroups (23 healthy controls and 23 asthma patients). [file Data_Sheet_1.docx]

Supplementary Material

TABLE S1 Clinical characteristics of the 46 BMI-matched subgroups (23 healthy controls and 23 asthma patients).

| Parameters | Healthy (n=23) | Asthmatic (n=23) | *P* value* |
| --- | --- | --- | --- |
| Age (years) | 43.8±23.3 | 44.0±21.2 | 0.979 |
| Sex, n (male:female) | 18:5 | 12:11 | 0.063 |
| BMI (kg/m^2^) | 22.3±2.7 | 23.6±3.1 | 0.158 |
| Smoking, n (yes:no) | 7:16 | 5:18 | 0.502 |

BMI: body mass index. *The data are presented as n (chi-square test) or as the mean±standard deviation (independent t-test). *P* <0.05 is considered significant between groups.

TABLE S2 The BLAST results of the most abundant OTUs (average RA of >1% in any group) in the airway bacterial microbiota of healthy controls and asthma patients.

| OTU ID | Taxonomy | Query Cover (%) | | Percent Identity (%) | |
| --- | --- | --- | --- | --- | --- |
| otu0 | *Streptococcus* sp. | 100 | 100 | |  |
| otu1 | *Neisseria* sp. | 100 | 100 | |  |
| otu2 | *Prevotella melaninogenica* | 100 | 100 | |  |
| otu3 | *Porphyromonas* sp. | 100 | 100 | |  |
| otu4 | *Gemella morbillorum* | 100 | 100 | |  |
| otu5 | *Streptococcus* sp. | 100 | 100 | |  |
| otu6 | *Haemophilus parainfluenzae* | 100 | 100 | |  |
| otu7 | *Porphyromonas endodontalis* | 100 | 100 | |  |
| otu8 | *Granulicatella* sp. | 100 | 100 | |  |
| otu9 | *Lautropia* sp. | 100 | 100 | |  |
| otu10 | *Fusobacterium* sp. | 100 | 100 | |  |
| otu12 | *Porphyromonas* sp. | 100 | 100 | |  |
| otu13 | *Streptococcus* sp. | 100 | 100 | |  |
| otu15 | *Prevotella pallens* | 100 | 100 | |  |
| otu16 | *Peptostreptococcus* sp. | 100 | 100 | |  |
| otu19 | *Moraxella* sp. | 100 | 100 | |  |
| otu21 | *Prevotella* sp. | 100 | 99.6 | |  |
| otu22 | *Veillonella* sp. | 100 | 100 | |  |
| otu53 | *Rothia mucilaginosa* | 100 | 100 | |  |

TABLE S3 The BLAST results of the most abundant OTUs (average RA of >1% in any group) in the airway fungal microbiota of healthy controls and asthma patients.

| OTU ID | Taxonomy | Query Cover (%) | Percent Identity (%) |
| --- | --- | --- | --- |
| otu0 | *Meyerozyma guilliermondii* | 100 | 100 |
| otu2 | *Candida palmioleophila* | 100 | 100 |
| otu3 | *Phialemonium dimorphosporum* | 100 | 100 |
| otu4 | *Candida albicans* | 100 | 100 |
| otu5 | *Aspergillus* sp. | 100 | 100 |
| otu6 | *Schizophyllum commune* | 100 | 100 |
| otu9 | *Aspergillus niger* | 100 | 100 |
| otu12 | *Malassezia restricta* | 100 | 100 |
| otu20 | *Epicoccum nigrum* | 100 | 100 |
| otu157 | *Pseudocercospora* sp. | 100 | 100 |

TABLE S4 Prevalence (P %) and RA (%) of the most abundant OTUs (RA of >1% in any group) in the airway bacterial microbiota of healthy controls and asthma patients.

| OTU ID | Species | Controls (n=26) | | Asthma (n=112) | | | *P* value |
| --- | --- | --- | --- | --- | --- | --- | --- |
|  |  | P | RA | P | RA |  | |
| otu0 | *Streptococcus* sp. | 100.00 | 7.64 | 100.00 | 7.54 |  | |
| otu1 | *Neisseria* sp. | 100.00 | 8.43 | 99.11 | 7.23 |  | |
| otu2 | *Prevotella melaninogenica* | 100.00 | 7.74 | 100.00 | 6.42 |  | |
| otu3 | *Porphyromonas* sp. | 96.15 | 1.91 | 96.43 | 4.12 | ** | |
| otu4 | *Gemellaceae* sp. | 100.00 | 3.75 | 97.32 | 2.59 | * | |
| otu5 | *Streptococcus* sp. | 96.15 | 1.66 | 99.11 | 1.85 |  | |
| otu6 | *Haemophilus parainfluenzae* | 100.00 | 4.34 | 94.64 | 3.15 |  | |
| otu7 | *Porphyromonas endodontalis* | 88.46 | 2.16 | 85.71 | 1.47 |  | |
| otu8 | *Lactobacillales* sp. | 96.15 | 1.65 | 98.21 | 1.87 |  | |
| otu9 | *Lautropia* sp. | 100.00 | 1.49 | 91.96 | 1.25 |  | |
| otu10 | *Fusobacterium* sp. | 100.00 | 2.27 | 99.11 | 2.28 |  | |
| otu12 | *Porphyromonas* sp. | 100.00 | 5.47 | 91.96 | 2.34 | * | |
| otu13 | *Streptococcus* sp. | 96.15 | 1.94 | 81.25 | 0.88 | *** | |
| otu15 | *Prevotella* sp. | 96.15 | 1.23 | 91.96 | 1.31 |  | |
| otu16 | *Peptostreptococcus* sp. | 92.31 | 1.36 | 92.86 | 1.25 |  | |
| otu19 | *Moraxella* sp. | 0.00 | 0.00 | 16.07 | 2.08 | * | |
| otu21 | *Prevotella* sp. | 76.92 | 0.30 | 92.86 | 1.18 | *** | |
| otu22 | *Veillonella parvula* | 100.00 | 2.06 | 98.21 | 1.22 | *** | |
| otu53 | *Rothia mucilaginosa* | 88.46 | 1.63 | 50.89 | 0.50 | *** | |

The *P* value represents the significant difference in the RA between healthy subjects and asthma patients. Statistical results of the RA between the two groups according to the Mann-Whitney U-test, *: *P*<0.05; **: *P*<0.01; ***: *P*<0.001.

TABLE S5 Prevalence (P %) and RA (%) of the most abundant OTUs (RA of >1% in any group) in the airway fungal microbiota of healthy controls and asthma patients.

| OTU ID | Species | Controls (n=29) | | Asthma (n=116) | | *P* value |
| --- | --- | --- | --- | --- | --- | --- |
|  |  | P | RA | P | RA |  |
| otu0 | *Meyerozyma guilliermondii* | 100.00 | 30.06 | 80.17 | 21.54 |  |
| otu2 | *Candida* sp. | 55.17 | 0.97 | 69.83 | 11.50 | *** |
| otu3 | *Sordariomycetes* sp. | 27.59 | 0.32 | 48.28 | 2.87 | ** |
| otu4 | *Candida albicans* | 68.97 | 1.45 | 79.31 | 3.71 | *** |
| otu5 | *Aspergillus* sp. | 44.83 | 2.27 | 43.97 | 1.59 |  |
| otu6 | *Schizophyllum commune* | 13.79 | 0.28 | 38.79 | 2.24 | ** |
| otu9 | *Aspergillus niger* | 34.48 | 0.34 | 62.93 | 2.75 | *** |
| otu12 | *Malassezia restricta* | 37.93 | 0.47 | 50.00 | 1.18 | * |
| otu20 | *Epicoccum nigrum* | 20.69 | 3.49 | 10.34 | 0.06 |  |
| otu157 | *Agaricomycetes* sp. | 0.00 | 0.00 | 49.14 | 1.60 | *** |

The *P* value represents the significant difference in the RA between healthy subjects and asthma patients. Statistical results of the RA between the two groups according to the Mann-Whitney U-test, *: *P*<0.05; **: *P*<0.01; ***: *P*<0.001.

TABLE S6 The interactions between airway bacterial/fungal microbiota and the genus *Moraxella* and *Moraxella* sp. otu19 in the networks for asthma and healthy subjects.

| Taxon1 | Taxon2 | Correlation | *P* value | Interaction |
| --- | --- | --- | --- | --- |
| *Moraxella* | *Veillonella* | -0.198207473 | 0.01 | co-exclusion |
| *Moraxella* sp. otu19 | *Meyerozyma guilliermondii* otu0 | 0.120874491 | 0 | co-occurrence |
| *Moraxella* sp. otu19 | *Agaricomycetes* sp. otu157 | 0.075120249 | 0 | co-occurrence |
| *Moraxella* sp. otu19 | *Candida* sp. otu2 | 0.081697657 | 0 | co-occurrence |
| *Moraxella* sp. otu19 | *Epicoccum nigrum* otu20 | 0.082128588 | 0 | co-occurrence |
| *Moraxella* sp. otu19 | *Sordariomycetes* sp. otu3 | 0.125495366 | 0 | co-occurrence |
| *Moraxella* sp. otu19 | *Aspergillus* sp. otu5 | 0.098396299 | 0 | co-occurrence |
| *Moraxella* sp. otu19 | *Schizophyllum commune* otu6 | 0.091797712 | 0 | co-occurrence |
| *Moraxella* sp. otu19 | *Aspergillus niger* otu9 | 0.119040992 | 0 | co-occurrence |
| *Moraxella* sp. otu19 | *Malassezia restricta* otu12 | 0.118164083 | 0 | co-occurrence |
| *Moraxella* sp. otu19 | *Streptococcus* sp. otu5 | -0.142984848 | 0 | co-exclusion |
| *Moraxella* sp. otu19 | *Lautropia* sp. otu9 | -0.140320267 | 0.01 | co-exclusion |
| *Moraxella* sp. otu19 | *Veillonella parvula* otu22 | -0.178816101 | 0.02 | co-exclusion |
